# Reversible cardiomyopathy in a patient with chronic myelomonocytic leukemia treated with decitabine/cedazuridine: a case report

**DOI:** 10.1186/s40959-023-00153-6

**Published:** 2023-01-18

**Authors:** Ankur Sheel, Junu Bae, Ashlee Asada, Gregory A. Otterson, Ragavendra R. Baliga, Kristin L. Koenig

**Affiliations:** 1 Department of Internal Medicine, The Ohio State University Wexner Medical Center, Columbus, OH 43210 USA; 2 College of Medicine, The Ohio State University, Columbus, OH 43210 USA; 3 Division of Oncology, Department of Internal Medicine, The Ohio State University and The Ohio State University Comprehensive Cancer Center, Columbus, OH 43210 USA; 4 Division of Cardiovascular Medicine, Department of Internal Medicine, Cardio-Oncology Center of Excellence, The Ohio State University Wexner Medical Center, OH, Columbus, OH 43210 USA; 5 Division of Hematology, Department of Medicine, The Ohio State University and The Ohio State University Comprehensive Cancer Center, Columbus, OH 43210 USA

**Keywords:** Decitabine/Cedazuridine, Chronic Myelomonocytic leukemia, Cardio-oncology, Cardiomyopathy

## Abstract

**Background:**

Hypomethylating agents (HMAs) have shown efficacy in the treatment of hematological malignancies and are indicated for the treatment of chronic myelomonocytic leukemia (CMML). While the HMA decitabine, in its intravenous formulation, has been used since 2006 for the treatment of CMML, use of its oral formulation has been limited by poor bioavailability due to first-pass metabolism by the enzyme cytidine deaminase. The dose of intravenous decitabine is limited by toxicities such as cardiomyopathy and heart failure. Therefore, cedazuridine was developed as an inhibitor of cytidine deaminase. Cedazuridine decreases the first-pass metabolism of oral decitabine allowing therapeutic levels to be achieved at lower doses, and thus, the novel oral combination of cedazuridine with decitabine was developed. While cardiomyopathy and heart failure are well-established adverse effects associated with intravenous decitabine alone, there to our knowledge there have been no documented incidences of reversible cardiomyopathy in the literature or in patients who participated in the phase 2 and phase 3 clinical trials of oral decitabine-cedazuridine.

**Case:**

This case study presents an 85 year-old Caucasian female with CMML who developed cardiomyopathy and heart failure with reduced ejection fraction after completing 5 cycles of therapy with decitabine/cedazuridine. Furthermore, her symptoms and cardiac function recovered upon discontinuation of the drug.

**Conclusions:**

We present an occurrence of reversible cardiomyopathy in a patient who completed 5 cycles of decitabine/cedazuridine, an oral combination therapy developed to enhance oral bioavailability of decitabine thereby limiting its adverse effects. As the decitabine/cedazuridine combination therapy rises in popularity due to its convenient oral formulation, more trials are needed to understand the prevalence of cardiomyopathy with this drug and to discover preventative strategies for cardiotoxic effects.

## Background

Hypomethylating agents (HMAs) incorporate into deoxyribonucleic acid / ribonucleic acid (DNA/RNA) during cell division and inhibit epigenetic silencing in malignant cells thus modifying epigenetic patterns and thereby altering gene expression [1]. This gene expression alteration is thought to contribute to the clinical activity of these agents [2]. HMAs have shown efficacy in the treatment of hematological malignancies and are indicated for the treatment of Myelodysplastic Syndrome (MDS), Acute Myeloid Leukemia (AML) and Chronic Myelomonocytic Leukemia (CMML) [3].

Decitabine and cedazuridine are nucleoside derivatives that operate synergistically to prevent DNA methylation, a biochemical process critical for regulating gene transcription. Following phosphorylation, decitabine triphosphate is directly incorporated into DNA, after which it irreversibly inhibits DNA methyltransferase [4]. While intravenous (IV) decitabine has been used since 2006 for the treatment of MDS, AML, and CMML, use of its oral formulation has been limited by poor bioavailability due to first-pass metabolism by the enzyme cytidine deaminase [5]. Thus high, toxic-level, doses of decitabine would be required in order to reach therapeutic levels when taken orally [6, 7]. Cedazuridine was developed as an inhibitor of cytidine deaminase, decreasing the first-pass metabolism of oral decitabine allowing therapeutic levels to be achieved at lower doses. The novelty of the combination of decitabine/cedazuridine (Inqovi, Astex Pharmaceuticals) is that the oral formulation has improved pharmacokinetics compared to decitabine alone, thus allowing for therapeutic levels to be achieved without the associated toxicities [5].

Cardiomyopathy and heart failure are known adverse effects of IV decitabine alone, with the incidence of peripheral edema occurring in approximately 25% of patients [8]. Moreover, in a phase 2 clinical trial of single agent IV decitabine for the treatment of CMML, the incidence of new onset Grade I-III and Grade IV/V cardiac events were 4.8 and 2.4%, respectively [9]. While cardiomyopathy and heart failure are well-established adverse effects associated with IV decitabine alone, there were no documented incidences of reversible cardiomyopathy in patients who participated in the phase 2 and phase 3 clinical trials of oral decitabine-cedazuridine [7, 8, 10, 11]. Furthermore, to our knowledge there have been no cases of this described in the literature. Here, we present a case of reversible non-ischemic cardiomyopathy in an 85-year-old female who was being treated with oral decitabine/cedazuridine for CMML.

## Case presentation

We present an 85 year-old Caucasian female with CMML but no significant past cardiac history. The patient was diagnosed with CMML by bone marrow biopsy. At the time of diagnosis, she was complaining of abdominal pain and weight loss. Her laboratory results were notable for severe left-shifted leukocytosis with white blood cells (WBC) 133 × 10^3^ K/uL (normal range 3.99–11.19 K/ul), an absolute monocytosis of 57.40 K/uL (normal range 0.22–0.87 K/ul), and thrombocytopenia with platelets 55 × 10^3^/uL (normal range 150–393 K/ul). Bone marrow biopsy demonstrated a hypercellular bone marrow (> 95%) with granulocytic hyperplasia, monocytosis, bilineage dysplasia, and a mild increase in blast cells (3%). AML and myeloproliferative neoplasm fluorescence in situ hybridization (FISH) and Next Generation Sequencing was negative for BCR-ABL1 translocation, JAK2, MPL, and CALR mutations. Of note the following somatic mutations were detected in the patient: CBL (L380P), SRSF2 (P95L) and TET2 (G1361D). The patient was started on hydroxyurea therapy with an initial response to therapy. However, 8 months into therapy, her total WBC count and monocytes began to increase from 9.77 × 10^3^ K/uL and 3.31 K/ul respectively to 48.54 10^3^ K/uL and 16.79 K/ul respectively. She also had difficulty tolerating increasing doses of hydroxyurea therapy secondary to persistent fatigue and dyspnea. Consequently, therapy was escalated to oral decitabine 35 mg/cedazuridine 100 mg (Inqovi) 1 tablet on days 1–5 of a 28-day cycle.

Of importance, this patient also has a remote history of ER/PR-positive HER2-negative metastatic left infiltrating ductal carcinoma first diagnosed 20 years prior to her CMML diagnosis. Her breast cancer was initially treated with lumpectomy and post operative local regional radiation therapy. She received 1.5 years of tamoxifen which was discontinued due to vaginal bleeding and was placed on annual surveillance. 10 years later, imaging demonstrated subpleural nodules and intrathoracic lymphadenopathy. Biopsy of one of the pleural nodules was consistent with metastatic breast cancer. She was started on palliative hormone therapy with letrozole. She never received cytotoxic chemotherapy for this disease, including treatment with anthracyclines or anti-HER-2 therapeutics.

After completing 5 cycles of therapy with decitabine-cedazuridine for her CMML, the patient presented to The Ohio State University Wexner Medical Center James Comprehensive Cancer Center with several days of cough, nausea, and emesis. Prior to this, she was tolerating the decitabine/cedazuridine well with no delays in therapy or dose reductions. On admission, laboratory results revealed WBC count of 7.44 × 10^3^/uL, hemoglobin of 8.5 g/dL (normal range 13.4 g/dL – 16.8 g/dL), and platelet count of 36 × 10^3^/uL. Hepatic function panel was significant for transaminitis with aspartate transaminase (AST) 216 U/L (normal range 10–39 U/L), alanine transaminase (ALT) U/L 652 (normal range 10–52 U/L) and total bilirubin of 1.4 mg/dL (normal range < 1.5 mg/dL). Creatinine and serum acetaminophen levels were within normal limits. Epstein Barr Virus (EBV) and Cytomegalovirus (CMV) polymerase chain reaction (PCR) and routine viral hepatitis panels were negative. In addition, testing showed brain natriuretic peptide (BNP) of 1687 pg/mL (normal range < 100 pg/mL), high sensitivity-troponin of 196 ng/L (normal range < 53 ng/L) and an associated tachycardia with a heart rate in the 120 range. Electrocardiogram (ECG) demonstrated sinus tachycardia with premature atrial complexes and nonspecific ST- and T-wave abnormalities. Chest x-ray revealed mild interstitial edema. Repeat high sensitivity troponins decreased after 1 hour and 8 hours to 176 ng/L and 130 ng/L, respectively. 2 weeks prior to admission her transaminases and bilirubin were within the normal range. Her medications on admission included daily acyclovir 800 mg, alendronate 70 mg, allopurinol 300 mg, gabapentin 300 mg and letrozole 2.5 mg. She was on Cycle 5 Day 17 of decitabine/cedazuridine at the time of presentation. Notably, she was not on any guideline-directed medical therapy for heart failure at the time of admission. Exam on admission was notable for non-distended jugular venous pulse, no appreciable crackles in bilateral lung fields and no lower extremity edema.

**Fig. 1:**
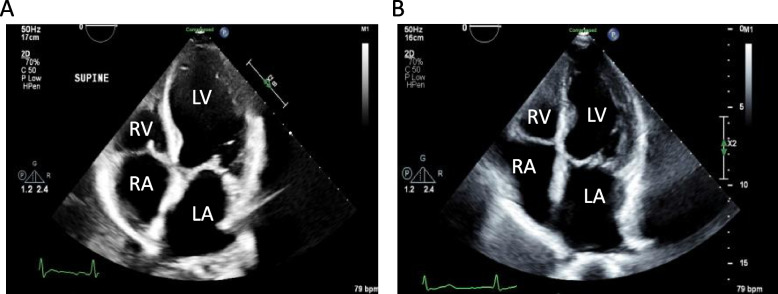
Transthoracic echocardiogram of patient in four chamber view during admission (**A**) and 2 months prior to admission (**B**) with representative chambers labeled

Given the BNP elevation and the interstitial edema seen on chest x-ray, as well as transaminitis that could suggest congestive hepatopathy, there was clinical concern for new cardiomyopathy and decompensated heart failure. In line with this, a transthoracic echocardiogram (TTE) showed new left ventricular enlargement with a new depressed left ventricular ejection fraction (LVEF) of 29% as well as chronic left atrial enlargement (Fig. 1A). No wall motion abnormality was noted on the TTE. Previous TTE 2 months prior to admission showed a preserved LVEF of 58% with severe left atrial enlargement (Fig. 1B).

Ischemic cardiomyopathy was thought to be less likely given lack of ischemic changes on electrocardiogram, mildly elevated troponins (which decreased) as well as lack of wall motion abnormalities on TTE. EBV, CMV and SARS-CoV-2 PCRs were negative at the time of admission and thyroid stimulating hormone (TSH) levels were within normal limits making viral myocarditis less likely and cardiomyopathy secondary thyroid dysfunction unlikely. Thus, the most likely explanation for her heart failure was felt to be drug-induced cardiomyopathy. Given the known adverse effect of cardiomyopathy with IV decitabine alone, there was concern that the development of her cardiomyopathy was secondary to decitabine/cedazuridine.

**Fig. 2:**
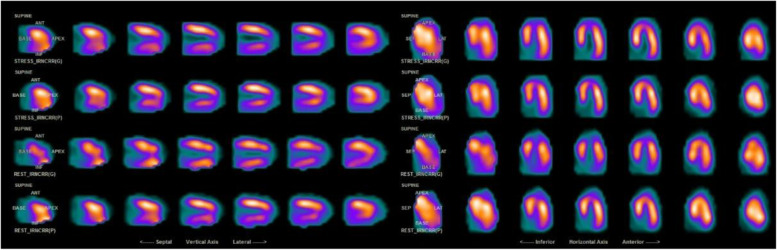
Representative image of patient’s nuclear stress test performed 2 months after admission demonstrating no radiographic evidence of ischemia

With aggressive diuresis, her cough, nausea, and vomiting markedly improved. Transaminitis improved, further suggesting congestive hepatopathy secondary to cardiomyopathy. By hospital day 6, she was feeling back to her baseline and was discharged on torsemide 10 mg, metoprolol succinate 25 mg, and spironolactone 25 mg daily. Decitabine/cedazuiridine was held during her hospitalization and on discharge. The patient was seen by a cardiologist 2 months after discharge and a pharmacologic nuclear stress test was performed demonstrating LVEF improvement to > 77% without evidence of ischemia or infarction (Fig. 2).

Her cardiologist and oncologist agreed to discontinue decitabine/cedazuridine indefinitely given this patient developed cardiomyopathy while taking this drug and recovered LVEF after its discontinuation. She instead resumed hydroxyurea for CMML disease control. Guideline-directed medical therapy was also continued given her American College of Cardiology / American Heart Association (ACC/AHA) Stage C, New York Heart Association (NYHA) Functional Class II cardiomyopathy.

## Discussion

In the case above, we describe an instance of acute decompensated heart failure secondary to non-ischemic cardiomyopathy in a patient with CMML treated with decitabine/cedazuridine with recovered ejection fraction upon cessation of the drug. While cardiomyopathy and heart failure are well documented toxicities associated with IV decitabine, this case describes the development of reversible non-ischemic cardiomyopathy in a patient who was treated with newer agent oral decitabine/cedazuridine.

The novelty of decitabine-cedazuridine arose from the fact that cedazuridine inhibits cytidine deaminase, the enzyme responsible for the first-pass metabolism of decitabine, therefore allowing decitabine to achieve therapeutic levels at lower doses. However, this case study suggests potential retained cardiotoxic effects of decitabine despite lower therapeutic dosing.

Overall, the oral formulation of decitabine-cedazuridine was found to have improved pharmacokinetics and bioavailability [5]. Following two independent randomized crossover trials (NCT02103478 and NCT03306264), this combination therapy was approved by the Food and Drug Administration (FDA) in July 2020 [11, 12]. Although the latter trial is still pending final results, the former has shown rare cardiotoxicity among its 80 participants, including instances of cardiac arrest (*n* = 3), cardiogenic shock (*n* = 1), myocarditis (n = 1), and pericardial effusion (n = 1) [11]. However, none of the participants in this clinical trial developed reversible cardiomyopathy.

Conversely, there are several accounts in the literature that describe reversible cardiotoxicity in patients taking IV decitabine as a single agent. The incidences of peripheral edema and heart failure with IV decitabine alone are 27 and 5%, respectively [13]. Furthermore, a 2017 retrospective study found a 16% incidence of grade 3 or greater cardiovascular toxicities in patients with AML treated with IV decitabine alone with normal renal function. The incidence of grade 3 or greater cardiovascular toxicities in patients with renal dysfunction, defined as a creatinine clearance of < 60 mL/min, increased to 33% [13]. Of note, our patient did not have any history of renal dysfunction.

Additionally, two separate case studies report cardiomyopathy associated with IV decitabine, with one recent report describing a delayed, irreversible cardiomyopathy in a 71-year-old patient whose symptoms arose after completing 10 cycles of IV decitabine, similar to the patient in this case [10, 14]. Previous studies have suggested that decitabine is metabolized into various subproducts and the exact elimination route of this metabolites remains unclear [15]. Given that toxicities of IV decitabine are increased in patients with renal disease, one proposed mechanism of decitabine toxicity involves decreased renal clearance. However, despite these reports, the mechanism of decitabine-induced cardiotoxicity remains unclear.

Here we present a case of acute, reversible cardiomyopathy in a patient with CMML who had completed 5 cycles of decitabine-cedazuridine. Given previous reports, it is likely that the underlying etiology of the cardiomyopathy observed in this case is directly related to decitabine toxicity. However, cedazuridine-related toxicity cannot be ruled out as it has never been studied as a standalone drug. One possible explanation for the toxicity observed in this patient involves activity levels of cytidine deaminase. Multiple studies have shown variable activity levels of cytidine deaminase in patients with both solid and liquid malignancies [16–18]. It is possible that in our patient, she had elevated activity or expression levels of cytidine deaminase such that drug levels present in decitabine/cedazuridine was not sufficient to inhibit metabolism of decitabine.

Alternatively, the patient’s mutational profile could have predisposed her to decitabine-cedazuiridne toxicity. While demethylating agent toxicity are not associated with CBL and SRSF2 mutations [19], higher abundance of TET2 mutations have been previously associated with increased sensitivity to hypomethylating agents in patients with MDS [20]. The mechanism driving this association remains unclear at this time but could be related to epigenetic instability during cell division as TET2 catalyzes the conversion of methylcytosine to 5-hydroxymethylcytosine [20]. Interestingly, our patient did have a TET2 mutation which may have put her at risk for toxicities associated with decitabine-cedazuridine. However, future studies are required to associate TET2 mutational burden with decitabine-cedauziridne toxicity and efficacy as TET2 could serve as a potential marker for drug toxicity.

Furthermore, viral myocarditis cannot be ruled out in our study as a possible etiology as this patient did not undergo cardiac magnetic resonance imaging (CMR) to assess for myocarditis. Despite the absence of this diagnostic test, infectious myocarditis was low on the differential since she had very mildly elevated troponin levels, an unremarkable ECG, a negative SARS-CoV-2 PCR and negative Epstein Barr Virus and Cytomegalovirus PCRs. As the decitabine/cedazuridine combination therapy rises in popularity due to its convenient oral formulation, more trials are needed to understand the prevalence of cardiomyopathy with this drug and to discover preventative strategies for cardiotoxic effects. We advise providers to monitor closely for cardiovascular and respiratory symptoms, even in patients who have already tolerated multiple cycles without such issues.

## Conclusions

This case is the first to demonstrate an example of cardiotoxicity in patient with CMML after completion of 5 cycles of decitabine/cedazuridine therapy in a patient without pre-existing cardiovascular disease. Cardiotoxicity is seen with IV decitabine alone, however the novelty of the combination of decitabine/cedazuridine was to mitigate risks associated with decitabine toxicity using an oral formulation. As the popularity of the oral formulation of decitabine/cedazuridine is increasing as treatment of various hematologic malignancies, we advise providers to monitor closely for cardiovascular and respiratory symptoms, even in patients who have already tolerated multiple cycles without such issues.

## Data Availability

All data generated or analyzed during this study are included in this published article.

## Abbreviations

ACC/AHA: American College of Cardiology / American Heart Association
AML: Acute myeloid leukemia
ALT: Alanine transaminase
AST: Aspartate transaminase
BNP: Brain natriuretic peptide
CMML: Chronic myelomonocytic leukemia
CMR: Cardiac magnetic resonance imaging
CMV: Cytomegalovirus
DNA: Deoxyribonucleic acid
EBV: Epstein Barr virus
ECG: Electrocardiogram
FDA: Food and Drug Administration
FISH: Fluorescence in situ hybridization
HMA: Hypomethylating agents
IV: Intravenous
LVEF: Left ventricular ejection fraction
MDS: Myelodysplastic syndrome
NYHA: New York Heart Association
PCR: Polymerase chain reaction
RNA: Ribonucleic acid
TSH: Thyroid stimulating hormone
TTE: Transthoracic echocardiogram
WBC: White blood cells
